# Loneliness Modulates Automatic Attention to Warm and Competent Faces: Preliminary Evidence From an Eye-Tracking Study

**DOI:** 10.3389/fpsyg.2019.02967

**Published:** 2020-01-17

**Authors:** Toshiki Saito, Kosuke Motoki, Rui Nouchi, Ryuta Kawashima, Motoaki Sugiura

**Affiliations:** ^1^Institute of Development, Aging and Cancer, Tohoku University, Sendai, Japan; ^2^Japan Society for the Promotion of Science, Tokyo, Japan; ^3^Department of Food Management, Miyagi University, Sendai, Japan; ^4^Smart Ageing Research Center, Aging and Cancer, Tohoku University, Sendai, Japan; ^5^International Research Institute of Disaster Science, Tohoku University, Sendai, Japan

**Keywords:** automatic attention, competence, loneliness, social cognition, warmth

## Abstract

Social connections are essential for human survival. Loneliness is a motivational factor for building and maintaining social connections. Automatic attention occurs with little cognitive effort and plays a key role in detecting biologically salient events, such as human faces. Although previous studies have investigated the effect of loneliness on social behavior, the effect of loneliness on automatic attention to human faces remains largely unknown. The present study investigated the effects of loneliness on automatic visual attention to warmth and competence facial information, which determines facial attraction. This study included 43 participants who rated warmth and competence facial information. Then, they engaged with the target-distractor paradigm in which they saw two house images at the top and bottom and indicated whether the images were identical. During the task, we presented two faces as distractors and measured visual attention toward the faces as automatic attention because participants did not have to attend to the faces. The results showed an interactive effect between subjective loneliness and facial information on automatic attention. Warm targets automatically captured the attention of people feeling relatively lonely, whereas competent targets automatically captured the attention of those who felt less lonely. These results suggest that loneliness adaptively influences automatic processing of social information.

## Introduction

Automatic attention is an adaptive tool for detecting and enhancing processing of salient events from an evolutionary perspective. Attention can be conceptualized to have two functions: voluntary (endogenous) attention, and automatic (exogenous) attention (Carretié, 2014). Voluntary attention is goal-driven and consciously directed toward the event or stimulus, while automatic attention is stimulus-driven and triggered by external events in the environment (Carretié, 2014). Automatic attention plays a key role in the efficient monitoring, detecting, and processing of biologically salient events that appear out of the current focus of attention, including fearful expressions (Hsu and Pessoa, 2007) pathogens (Van Hooff et al., 2014), and delicious-looking foods (Motoki et al., 2018).

The saliency of an event depends on an individual’s state, such as loneliness. Loneliness is the negative experience of a discrepancy between the desired and achieved personal network of relationships (Maner et al., 2007; de Jong Gierveld et al., 2016) and is related to negative emotional experiences (Hawkley and Cacioppo, 2010). Many studies have demonstrated that loneliness increases attention to social information, especially social threads, using various experimental methodologies such as electroencephalogram (Cacioppo et al., 2015, 2016) and eye-tracking (Bangee et al., 2014). Additionally, it is known that loneliness causes hypervigilance to visual cues about social information (e.g., Bangee et al., 2014) as well as auditory cues about social information (i.e., voice, Shin and Kim, 2019). Although it seems obvious that loneliness increases attention to social stimuli, the effects of loneliness on automatic visual attention to social information remain unclear. The abovementioned studies have addressed these effects on selective attention using Stroop (Cacioppo et al., 2015, 2016; Shin and Kim, 2019) and free-viewing (Bangee et al., 2014) tasks. However, other studies investigating the effects of loneliness on attention only measured voluntary attention (Gardner et al., 2005; Lodder et al., 2016) because the participants consciously paid attention to social information. Given the unique role that automatic attention plays in adaptive behaviors (Carretié, 2014), it is important to examine whether loneliness would also influence automatic visual attention to social information, such as faces. The human face presents perhaps the most primary form of social information because it reveals various indicators such as age, gender, race, mood, intentions, and focus (Bruce and Young, 1986). Indeed, people automatically make social judgments when seeing faces even if they do not expect to interact with the individual (Winston et al., 2002). Additionally, the manner in which people see faces can influence behaviors such as preference (Shimojo et al., 2003). Thus, the present study investigated the effects of loneliness on automatic visual attention to faces because faces contain important social information. In the current study, we set up the first hypothesis:

H1: Lonely people pay more automatic attention to faces compared to those who are less lonely.

Although loneliness promotes attention to faces, this behavior may depend on one’s impression of the face, such as warmth and competence. People automatically evaluate faces based on two fundamental dimensions, valence (warmth) and dominance (competence) (Oosterhof and Todorov, 2008; Saito et al., 2019), and these two facial impressions might influence automatic attention differently based on the level of loneliness. From an evolutionary perspective of a fundamental motivational framework (Griskevicius and Kenrick, 2013; Motoki and Sugiura, 2017), loneliness modulates the saliency of information. However, no studies have investigated whether loneliness also modulates automatic attention to faces based on the two fundamental dimensions of facial evaluation (i.e., warmth and competence). It is possible that warmth information is more salient than competency information to lonely people because affiliation, which is a fundamental motive when forming and maintaining cooperative alliances, is motivating. Indeed, lonely people show greater motivation for reconnecting with others (de Jong Gierveld et al., 2016). For example, lonely people tend to preferentially recall events related to social interactions when they are asked to recall events after reading another person’s diary. On the other hand, less lonely people do not show this type of behavior (Gardner et al., 2005). Moreover, socially excluded people pay more attention to signs of social acceptance (DeWall et al., 2009; Xu et al., 2015) and show a greater ability to distinguish between genuine and deceptive smiles (Bernstein et al., 2008). Therefore, warm faces would capture more automatic attention from lonely people.

In contrast, competent faces are more salient to less lonely people. In some cases, the perception of competence is more important than the perception of warmth (Wojciszke, 2005; Wojciszke and Abele, 2008; Cuddy et al., 2011). For example, people who seek affective information (e.g., an advertisement focusing on the pleasantness of the product) place more significance on the perception of warmth whereas people who seek cognitive information (e.g., an advertisement focusing on attributes such as ingredients or the manufacturing of the product) place more significance on the perception of competence (Aquino et al., 2016). The need for cognition is indirectly correlated with low levels of loneliness; two traits associated with loneliness (self-esteem and social anxiety) are associated with the need for cognition (Osberg, 1987). Specifically, self-esteem and low anxiety levels are both positively correlated with the need for cognition and correlated with low levels of loneliness (Al Khatib, 2012; Lim et al., 2016). These findings suggest that less lonely people seek competence rather than warmth in others. Previous studies of fundamental human motivation have shown that acquiring higher status in a group (status motive) is a primary concern after fulfilling the need for affiliation (Kenrick et al., 2010) because higher status enables people to access desirable mates, food, and resources more easily (Crosier et al., 2012; O’Connor et al., 2014). If an individual wants to acquire status within a group, then competence rather than warmth in others becomes important because perceived competence is highly correlated with status (Fiske et al., 2007). Considering the status motive (Kenrick et al., 2010), it is adaptive for people feeling less lonely to detect a competent person because the person who potentially acquires a higher status can establish and maintain a network of alliances, which may require initially gaining status or acquiring territory. Moreover, given that the motivation to mate is subordinate to the motivation for status, it is adaptive to identify a competent person who may be a desired mating partner for opposite-sex perceivers and a strong competitor for own-sex perceivers. Indeed, individuals with mating-related but not affiliation-related motivation have been shown to pay more attention to high-status men (DeWall and Maner, 2008). Thus, competent faces would capture more automatic attention from less lonely people. Given the role of automatic attention, which is the detection of biologically salient events, we set up the second hypothesis.

H2a: A greater level of loneliness promotes automatic attention to warm faces.
H2b: Less loneliness promotes automatic attention to competent faces.

This study sought to clarify how the internal state of loneliness influences the rapid and efficient processing of social information by simultaneously presenting human faces and non-social images, such as a house, and using an eye-tracking device to examine whether loneliness promotes automatic (task irrelevant) attention to faces and whether loneliness promotes automatic attention only to warm faces. We set up two hypotheses. The first hypothesis is that people feeling more lonely pay more automatic attention to faces, as compared to those who are less lonely. The second hypothesis is that the degree of loneliness modulates the extent of automatic attention; a greater degree of loneliness promotes automatic attention to warm faces whereas a lesser degree of loneliness promotes automatic attention to competent faces.

## Materials and Methods

### Participants

Forty-four Tohoku University undergraduates and graduates (22 men and 22 women) participated. One participant was excluded from the analysis due to deficiencies in the eye-tracking calibration. Thus, there were 43 participants (21 men and 22 females, mean age: 21.00, *SD* = ± 1.90 years). Participants received about $10 for their participation.

### Design

The present study included three independent continuous variables: (1) warmth rating of faces, (2) competency rating of faces, and (3) subjective rating of loneliness. The primary outcome was automatic attention toward faces.

### Apparatus

The present study used the Tobii Pro X2-60 (60 Hz; Tobii Technology, Stockholm, Sweden) to monitor eye movements; this system does not require anything to restrict head movement (e.g., a chin rest) The stimuli were shown on an LCD monitor with a resolution of 1920 × 1080. The distance between the participant and the display was approximately 60 cm. Participants were calibrated using a nine-point calibration in the Tobii studio. During the eye-tracker task, participants were instructed to make their best effort not to move their heads.

### Materials

#### Loneliness Measure

The UCLA Loneliness Scale (version 3) includes 20 items to measure the degree of perceived social isolation (Russell, 1996). Participants rated how often they have experiences that made them feel isolated, such as, “My social relationships are superficial,” and, “I feel alone.” We used a Japanese version of the UCLA Loneliness Scale (version 3), which has been confirmed to have high reliability and validity in a Japanese sample (Masuda et al., 2012).

#### Facial Stimuli

We created 163 three-dimensional male faces using software (FaceGen Modeller 3.14) after referring to a previous study (Oosterhof and Todorov, 2008; Saito et al., 2017; Motoki et al., 2019). The faces were rated by participants of the present study with regard to warmth (“How warm is this person?”), competence (“How competent is this person?”), and attractiveness (“How attractive is this person?”). They answered each question on a seven-point Likert scale from 1 to 7 (cold-warm, incompetent-competent, and unattractive-attractive). Each question was blocked and they took a short rest after answering 50 questions. This face-rating task was presented using PsychoPy (Peirce, 2007) and, based on the ratings of each participant, 80 face stimuli in which warmth and competence had minimal correlations were selected for each participant. Thus, a different set of facial stimuli was used for each participant. The correlation between warmth and competence was minimized as much as possible to avoid the multicollinearity problem because the analyses included warmth and competence ratings as independent variables (*r* = 0.166). The selection procedure for the facial stimuli was performed as follows. First, the faces were categorized as warm and competent, warm but incompetent, cold but competent, and cold and incompetent. Faces that received a score of 4 (scale midpoint) or higher on the warmth scale were categorized as warm faces and those that scored below four-points were categorized as cold faces. Similarly, faces that received a score of 4 or above on the competence scale were categorized as competent faces and those rated as less than 4 were categorized as incompetent faces. Then, 20 faces were randomly selected from each category; the mean ratings for each category are presented in Table 1. To determine any differences for each rating among the four categories, the ratings were assessed with one-way analysis of variance (ANOVA) tests. There were significant main effects of the different categories on each rating (warmth: *F*[1.47,162.50) = 316.36, *p* = 0.000, ${\text{η}}_{\text{p}}^{2}$ = 0.88, competence: *F*[1.47,62.21) = 299.59, *p* = 0.000, ${\text{η}}_{\text{p}}^{2}$ = 0.87, and attractiveness: *F*[2.26,94.76) = 79.01, *p* = 0.000, ${\text{η}}_{\text{p}}^{2}$ = 0.65). Subsequent multiple comparison analyses revealed that stimuli categorized as warm were rated as warmer than those categorized as cold. Additionally, stimuli categorized as competent were rated as more competent than those categorized as incompetent (Appendix Table A1). Thus, it was confirmed that the four categories were successfully divided based on the participant ratings.

**TABLE 1:** Results of the face- rating task.

**Stimuli category**	**Warmth**		**Competence**		**Attractiveness**	
		
	***Mean***	***SE***	***Mean***	***SE***	***Mean***	***SE***
Warm and competent	4.83	0.07	4.85	0.07	4.11	0.09
Warm but incompetent	4.53	0.08	3.03	0.07	3.44	0.08
Cold but competent	2.84	0.07	4.69	0.07	3.63	0.07
Cold and incompetent	2.67	0.08	2.82	0.06	3.03	0.07

*SE represents standard error. Ratings of warmth on a scale of 1–7 (‘Cold’ to ‘Warm’). Ratings of competence on scale of 1–7 (‘incompetent’ to ‘competent’). Ratings of attractiveness on scale of 1–7 (‘unattractive’ to ‘attractive’).*

#### House Stimuli

The present study displayed 10 house images used in a previous study (Motoki et al., 2018). The images contained three elements: a house, a yard, and the sky.

### Procedure

**FIGURE 1:**
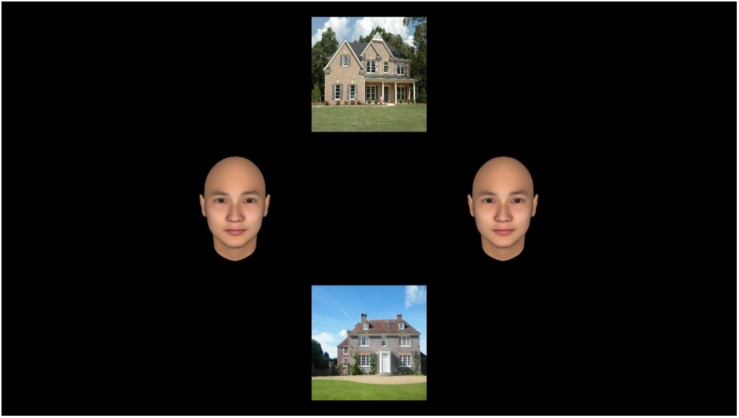
An example of the face-house task. Participants indicated whether the target images (house) located top and bottom were identical or not. Two identical faces were located side by side as distracters. Total viewing time of the faces was measured as automatic attention.

Groups of two to four participants were placed in a room that accommodated a maximum of 10 people. After obtaining informed consent from each participant, all participants completed the UCLA Loneliness Scale (Russell, 1996). Because there were only two laptops available for the ratings task and all participants could not perform the task at the same time, half of participants did so before and the remainder did so after completing the face-rating task. The loneliness rating (*t*[41] = 0.549, *p* = 0.581, *n.s.*) did not differ between groups; therefore, we concluded that task order did not affect the degree of loneliness. Then, the participants performed a filler task that was unrelated to the current study for 10 min. After that, we asked the participants to perform a face-house task, which employed the concurrent but distinct target-distractor paradigm (Carretié, 2014). First, a fixation cross appeared for 2 s. Then, two house images and two identical facial images appeared on the screen (Figure 1). We presented the house images at the top and bottom of the screen as the targets and the facial images on the left and right sides of the screen as distractors using Tobii software (ver. 3.3.2; Tobii Technology). The participants were required to indicate whether the targets (houses) were identical or different within each trial by either pressing the F key (same) or the J key (different). Additionally, we asked them to respond as soon as they knew the correct answer. A fixation cross appeared for 1 s between trials. The task was divided into two sessions of 40 trials each, and there was a short rest between sessions. There were 20 same house trials and 20 different house trials in each session. There was a total of 80 trials and the participants encountered 20 faces from each of the four categories (warm and competent, warm but incompetent, cold but competent, cold and incompetent; 80 in total). The order of the trials and the sessions were counterbalanced.

### Statistical Analyses

All statistical analyses were conducted using R software (R Core Team, 2017). We used the lme4 package in Bates et al. (2015) for the generalized linear mixed model (GLMM) and *p*-values for each effect were obtained based on Satterthwaite’s approximation using the lmerTest package (Kuznetsova et al., 2017). The statistical power of each analysis was retrospectively assessed using the smir package (Green and MacLeod, 2016). Then, one-tailed *p*-values for the two hypotheses were calculated because these hypotheses posited the specific directions of the differences.

To assess automatic attention to human faces in each trial, the screen was divided into two areas of interest (AOI); i.e., houses (target) and faces (distractors). These AOI were defined as follows: houses (target), which were the outlines of both top and bottom house images, and faces (distractors), which were the outlines of both sides of face images. Total fixation times on the houses (target) and faces (distractors) were measured using eye tracking. Automatic attention was defined as total fixation time on faces (distractors) because participants were not required to attend to the distractors. These statistical procedures were described in a previous study (Motoki et al., 2018).

The total viewing time of faces was entered into the GLMM as a dependent variable and subject was entered as a random effect to control for repeated measures. Five fixed effects, including the warmth and competence ratings of the facial stimuli that were rated prior to the face-house task, were also entered into the model. Subjective loneliness, which was also rated prior to the face-house task, and the interactions between warmth and loneliness and between competence and loneliness were also assessed.

Six covariates (i.e., attractiveness, brightness of the images, total house viewing time in each trial, trial condition [same/different], age of the participant, and sex of the participant) were entered into the subsequent model to account for potential effects from other factors. Attractiveness was rated by each participant prior to the face-house task and entered into the model because attractiveness captures attention (Sui and Liu, 2009). The brightness of images was calculated using the rgb2gray and mean2 functions of MATLAB 2017a^1^ and was entered into the model because brightness captures attention (e.g., Turatto and Galfano, 2000). Total house viewing time and trial condition were entered because trial difficulties that might be reflected in these variables could affect gaze behavior and viewing time for both the targets and distractors. Age and sex were entered to reduce individual differences. All variables were standardized, except for nominal variables, trial condition (same: 0.5, different: −0.5), and sex of the participants (male: 0.5, female: −0.5).

## Results

Table 2 presents the descriptive statistics of the following variables: mean score for loneliness, reaction time (ms, which is the average time for one trial in the face-house task), viewing time of the houses and faces (ms), accuracy of the task performance (%), and disrupt rate (%), which was the percentage of trials in which participants looked at the distractors.

**TABLE 2:** Descriptive statistics.

**Variable**	***Mean***	***SE***
Loneliness	38.40	1.69
Response time (ms)	990.52	10.96
Viewing time of houses (ms)	569.38	6.16
Viewing time of faces (ms)	86.63	4.26
Accuracy (%)	98.78	1.67
Disrupt rate (%)	20.84	6.19

*Viewing Time of Houses is an average viewing time in trials in which participants looked at houses. Viewing Time of Faces is an average viewing time in trials in which participants looked at distractors. Accuracy is the percentage of trials in which participants responded correctly to the face-house task. Disrupt Rate is the percentage of trials in which participants looked at the distractors.*

First, a GLMM analysis was conducted to assess the effects of perceived traits (warmth and competence) and subjective loneliness on automatic attention. There was no main effect of loneliness on automatic attention to faces (β = 0.078, *z* = 1.231, *p* = 0.112, *n.s.*). This result indicates that loneliness did not promote automatic attention to faces regardless of the facial information, so the first hypothesis positing that people feeling relatively lonely would pay more automatic attention to faces, as compared to those who are less lonely was not supported. More importantly, there were significant interactions between perceived warmth and subjective loneliness (β = 0.028, *z* = 1.689, *p* = 0.046) and between perceived competence and subjective loneliness (β = −0.039, *z* = −2.381, *p* = 0.009). The full results of this analysis are presented in Appendix Table A2.

Analyses including the other covariates produced similar results. The marginal main effect of perceived warmth on automatic attention was significant (β = 0.031, *z* = 1.841, *p* = 0.033). The main effects of perceived competence and subjective loneliness were not significant (β*s* = 0.007, 0.061, *zs* = 0.419, 0.913, *ps* = 0.337, 0.183, *n.s.*). However, the interactions between perceived warmth and subjective loneliness (β = 0.027, *z* = 1.669, *p* = 0.048) and between perceived competence and subjective loneliness (β = −0.039, *z* = −2.428, *p* = 0.008) were significant. The complete results of this analysis are presented in Appendix Table A3.

**FIGURE 2:**
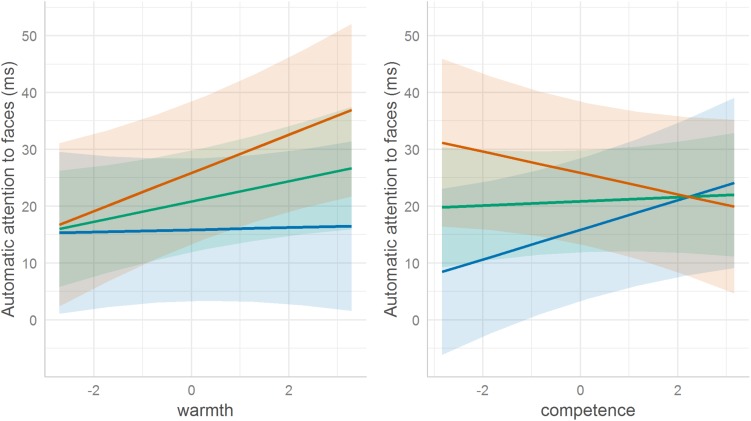
The interaction between the warmth evaluation and subjective loneliness **(left)** and the interaction between the competency evaluation and subjective loneliness **(right)**. The red line represents a high degree of loneliness (+ 1 SD). The green line represents average loneliness. The blue line represents a low degree loneliness (–1 SD). Shaded regions indicate 95% confidence intervals.

The present study also conducted a simple slope analysis to interpret each interaction (Figure 2). First, the simple slopes for the association between automatic attention and perceived warmth were tested for low loneliness (−1 SD below the mean) and high loneliness (+ 1 SD above the mean). The analysis showed that people feeling relatively lonely automatically paid attention to the warm targets (β = 0.058, *z* = 2.527, *p* = 0.006), whereas people feeling less lonely did not (β = 0.004, *z* = 0.153, *p* = 0.439, *n.s.*). A *post hoc* power analysis revealed high statistical power for detecting the simple effect of warmth when the loneliness rating was high (power = 0.80). On the other hand, when the loneliness rating was low, statistical power was low (power = 0.10). Although the statistical power was low for some of the analyses, it should be noted that *post hoc* power analyses, such as those performed in our study, may be misleading. According to Zumbo and Hubley (1998), it is nonsensical to make power calculations after a study has been conducted and a statistical decision has been made because there is a one-to-one correspondence between power and the *p*-value of any statistical test (Ellis, 2010). Thus, although the *post hoc* power analysis showed that the statistical power of the study was low, the study may not have underpowered.

The simple slopes for the association between automatic attention and perceived competence were tested for low and high loneliness. The analysis showed that people feeling less lonely paid significantly more attention to competent targets (β = 0.047, *z* = 2.001, *p* = 0.023), whereas those feeling relatively more lonely did not (β = −0.032, *z* = −1.324, *p* = 0.093). A *post hoc* power analysis revealed moderately high statistical power for detecting the simple effect of competence when the loneliness rating was low (power = 0.70). When the loneliness rating was high, power was low (power = 0.50).

## Discussion

The present study investigated how two fundamental impressions of faces, warmth and competence, and subjective loneliness would affect automatic attention. The results showed that warm targets captured the automatic attention of relatively lonely people, whereas competent targets captured the automatic attention of less lonely people. This result supports our hypothesis 2a and 2b that the degree of loneliness modulates the extent of automatic attention to warm and competent faces.

Our result that people who felt relatively lonely automatically paid attention to warm faces is consistent with the regulatory model of belonging need (Pickett and Gardner, 2005). According to the model, individuals monitor their level of social inclusion and maintain it at an acceptable level in the same way as they maintain basic needs (e.g., food, water, and sleep). When the sociometer or other assessment mechanism indicates that one’s state of belonging is unsatisfactory, the regulatory system becomes activated to monitor social information that may provide cues to belonging and inclusion. Indeed, people feeling deficient in social connections show a greater tendency to attend to social information, such as in the environment, compared with those who have a satisfactory level of acceptance (Gardner et al., 2005). The current study indicates that the monitoring system works automatically and that the system can monitor less salient information, such as perceived warmth, rather than salient information, such as a smiling face.

The adaptive change in the direction of automatic attention to faces is consistent with a goal systems framework (Kruglanski et al., 2002). According to this framework, activation of a goal, even unconsciously, leads people to present behavior to accomplish the goal. Indeed, people who are primed to activate a prestige goal chose a higher-priced option than do people primed to activate a thrift goal (Chartrand et al., 2008). Furthermore, when people are primed with the goal of reducing physical coldness, they prefer socially warm activities rather than control activities (Zhang and Risen, 2014). Therefore, it may be that the goal of the lonelier participants in our study was to connect with people they perceived as warm. Looking at warm faces is a useful strategy for connecting with warm people because a direct gaze gives the receiver a better impression of the sender (Ewing et al., 2010; Khalid et al., 2016). Conversely, the goal of less lonely participants may have been to acquire a higher status (Griskevicius and Kenrick, 2013), given that high status in a group (status motive) is a primary concern after fulfilling the need for affiliation (Kenrick et al., 2010). For these participants, directing their gaze toward competent faces is a useful strategy because building a good relationship with a higher status person may raise one’s own status.

Our findings are not consistent with the hypothesis that lonely people pay more automatic attention to faces than do less lonely people. Gardner et al. (2005) found that higher levels of loneliness were correlated with attention to social information. However, hypervigilance to social information depends on the emotional valence of the information. DeWall et al. (2009) reported that participants threatened with exclusion paid more attention to social information conveying social acceptance (smiling faces) than to that conveying social disapproval (angry and sad faces). These findings suggest that loneliness increased attention to positive but not negative social information. Our finding that lonely people paid more attention to warm faces than to faces in general is consistent with DeWall et al. (2009). However, previous studies have found that loneliness increased attention to negative social information. For example, Shin and Kim (2019) found that lonely people showed heightened attention to a negative vocal tone that signaled social threat. Thus, it may be important to elaborate when lonely people pay more attention to social acceptance versus social threats to further elucidate the role of loneliness in social circumstances.

In our results, we observed the main effect of warmth but not competence on automatic attention. Although perception is composed principally of warmth and competence information, previous studies have suggested that the warmth information is primary (Engell et al., 2007; Todorov et al., 2008). Trustworthiness (warmth) judgments after a 100-ms exposure to a target face is highly correlated with judgments made without time constraints compared to competent judgments (Willis and Todorov, 2006). These results suggest that people can make these judgments in a short time and that warmth judgments are made faster than other judgments. From an evolutionary perspective, the primacy of warmth makes sense because another person’s intent for good or ill will is more important to survival than whether the other person can act on those intentions (Fiske et al., 2007). Given these findings, it is reasonable to infer that warmth was generally more significant for capturing automatic attention than competence. On the other hand, the bias toward information about warmth or competence is influenced by deliberative aspects, such as the social interaction perspective (Wojciszke and Abele, 2008). According to these authors, warmth is more important than competence in cases of individuals who are not close to one another whereas competence is more important than warmth in cases of close friends and the self (Wojciszke and Abele, 2008). In the present study, automatic attention to warmth and competence information was modulated by loneliness. Thus, although warmth information was more important in general, the importance of warmth and competence information might depend on an individual’s state, such as loneliness, in some cases.

This study had several limitations. First, it was difficult to conclude a causal relationship between loneliness and eye movement behavior. Thus, a direct manipulation of loneliness, such as with the Cyberball paradigm (Williams et al., 2000), will be important to confirm the causal relationship. Second, our sample size may have affected the results. The study included 43 subjects, which was similar to the sample size of a previous study (*n* = 46) that used eye-tracking methodology to examine the effects of social exclusion on attention to faces (DeWall et al., 2009). Although the observed power of the main findings was not particularly low (the interaction of competence and loneliness was high [80%] and that of warmth and loneliness was moderately low [40%]), it would have been ideal to calculate the sample size based on the effect size of the previous relevant study prior to observing outcomes. Thus, a future study with an appropriate sample size based on the results of the present study will strengthen the reliability of our findings. Finally, the facial stimuli used in the present study were relatively artificial. Although artificial faces are advantageous in that it is possible to control extraneous factors such as hairstyle and lighting, these are not faces people would normally interact with. Because the concept of loneliness was the primary focus of the present study, the use of artificial faces may have decreased the ecological validity. Therefore, future studies using the same procedure should include more naturalistic faces as stimuli.

The present results provide new insight into automatic attention. Prior studies showed that salient stimuli (e.g., emotional faces and delicious-looking foods) capture automatic attention (Carretié et al., 2012; Motoki et al., 2018). Our study suggests that saliency of social stimuli is dependent on an individuals’ state, such as feeling lonely. Indeed, there is a modulatory effect of individual state-trait characteristics on automatic attention. For example, patients with generalized anxiety disorder present greater automatic attention to negative distractors than do healthy people (MacNamara and Hajcak, 2010). Therefore, future studies should consider the level of saliency changes based on individual states.

## Data Availability Statement

The datasets generated for this study are available on request to the corresponding author.

## Ethics Statement

This study was approved by the Ethical Committee of the School of Medicine at Tohoku University and was conducted in accordance with the Declaration of Helsinki. All participants in this study joined voluntarily and provided written informed consent prior to the participation.

## Author Contributions

TS, KM, RN, and MS designed and developed the study protocol. TS and KM conducted the study and collected the data. TS analyzed the data and wrote the manuscript. All authors interpreted the data, and read and approved the final manuscript. KM, RN, RK, and MS provided critical revisions.

## Conflict of Interest

The authors declare that the research was conducted in the absence of any commercial or financial relationships that could be construed as a potential conflict of interest.

## Footnotes

1 www.mathworks.com

## Appendix

**TABLE A1:** Results of multiple comparison analyses of the stimuli ratings between each category.

**Ratings**	**Comparisons**			**Difference**	***t*-Value**	***p*-Value**
**Warmth rating**						
	Warm-competent	>	Warm-incompetent	0.29	4.90	0.0000
		>	Cold-competent	1.99	19.60	0.0000
		>	Cold-incompetent	2.16	20.31	0.0000
	Warm-incompetent	>	Cold-competent	1.70	16.19	0.0000
		>	Cold-incompetent	1.87	17.95	0.0000
	Cold-competent	>	Cold-incompetent	0.17	6.30	0.0000
**Competence rating**						
	Warm-competent	>	Warm-incompetent	1.81	17.35	0.0000
		>	Cold-competent	0.16	4.18	0.0000
		>	Cold-incompetent	2.03	20.75	0.0000
	Cold-competent	>	Warm-incompetent	1.65	15.44	0.0001
		>	Cold-incompetent	1.87	19.06	0.0000
	Warm-incompetent	>	Cold-incompetent	0.22	4.14	0.0002
**Attractiveness rating**						
	Warm-competent	>	Warm-incompetent	0.67	8.90	0.0000
		>	Cold-competent	0.48	6.89	0.0000
		>	Cold-incompetent	1.08	11.18	0.0000
	Warm-incompetent	<	Cold-competent	–0.19	3.76	0.0005
		>	Cold-incompetent	0.41	7.04	0.0000
	Cold-competent	>	Cold-incompetent	0.60	8.75	0.0000

*df = 42. Reported *p*-values were corrected using the Shaffer’s modified Sequentially Rejective Bonferroni Procedure.*

**TABLE A2:** Full results of the GLMM analyses without covariates.

**Random effects**	**Name**	***SD***	**Variance**		
Participant	(Intercept)	0.403	0.163		
Residual		0.915	0.838		

**Fixed effects**	**Estimated β**	***SE***	***t*-Value**	***p*-Value**	**Power**

(Intercept)	0.000	0.063	–0.007	0.497	
Warmth	0.024	0.016	1.496	0.067	30%
Competence	–0.005	0.016	–0.324	0.373	20%
Loneliness	0.078	0.063	1.231	0.113	20%
Warmth × Loneliness	0.028	0.016	1.689	0.046	70%
Competence × Loneliness	–0.039	0.016	–2.381	0.009	90%

*Number of observations was 3440 and there were 43 participants.*

**TABLE A3:** Generalized linear mixed model analysis of the effects of perceived traits and loneliness on automatic attention with covariates.

**Random effects**	**Name**	***SD***	**Variance**		
Participant	(Intercept)	0.173	0.416		
Residual		0.834	0.913		

**Fixed effects**	**Estimated β**	***SE***	***t*-Value**	***p*-Value**	**Power**

(Intercept)	0.015	0.067	0.230	0.409	
Warmth	0.031	0.017	1.841	0.033	60%
Competence	0.007	0.017	0.419	0.338	10%
Loneliness	0.061	0.067	0.913	0.183	20%
Warmth × Loneliness	0.027	0.016	1.669	0.048	40%
Competence × Loneliness	–0.039	0.016	–2.428	0.008	80%

**Covariates**	**Estimated β**	***SE***	***t*-Value**	***p*-Value**	

Attractiveness	–0.029	0.019	–1.566	0.059	
Brightness	–0.022	0.016	–1.413	0.079	
Total house viewing time	0.058	0.018	3.217	0.001	
Trial condition	–0.027	0.031	–0.882	0.189	
Age	–0.002	0.066	–0.031	0.488	
Sex	0.190	0.133	1.433	0.080	

*The study included 3440 observations for 43 participants.*

## References

[B1] Al Khatib S. A. (2012). Exploring the relationship among loneliness, self-esteem, self-efficacy and gender in United Arab Emirates college students. *Eur. J. Psychol.* 8 159–181. 10.5964/ejop.v8i1.301

[B2] Aquino A. Haddock G. Maio G. R. Wolf L. J. Alparone F. R. (2016). The role of affective and cognitive individual differences in social perception. *Pers. Soc. Psychol. Bull.* 42 798–810. 10.1177/0146167216643936 27460272

[B3] Bangee M. Harris R. A. Bridges N. Rotenberg K. J. Qualter P. (2014). Loneliness and attention to social threat in young adults: findings from an eye tracker study. *Pers. Individ. Dif.* 63 16–23. 10.1111/sjop.12436 29516516

[B4] Bates D. Mächler M. Bolker B. Walker S. (2015). Fitting linear mixed-effects models using lme4. *J. Stat. Softw.* 67 1–48. 10.18637/jss.v067.i01

[B5] Bernstein M. J. Young S. G. Brown C. M. Sacco D. F. Claypool H. M. (2008). Adaptive responses to social exclusion: social rejection improves detection of real and fake smiles. *Psychol. Sci.* 19 981–983. 19000206 10.1111/j.1467-9280.2008.02187.x

[B6] Bruce V. Young A. (1986). Understanding face recognition. *Br. J. Psychol.* 77 305–327. 3756376 10.1111/j.2044-8295.1986.tb02199.x

[B7] Cacioppo S. Balogh S. Cacioppo J. T. (2015). Implicit attention to negative social, in contrast to nonsocial, words in the Stroop task differs between individuals high and low in loneliness: evidence from event-related brain microstates. *Cortex* 70 213–233. 10.1016/j.cortex.2015.05.032 26195152

[B8] Cacioppo S. Bangee M. Balogh S. Cardenas-Iniguez C. Qualter P. Cacioppo J. (2016). Loneliness and implicit attention to social threat: a high-performance electrical neuroimaging study. *Cogn. Neurosci.* 7 138–159. 10.1080/17588928.2015.1070136 26274315

[B9] Carretié L. (2014). Exogenous (automatic) attention to emotional stimuli: a review. *Cogn. Affect. Behavi. Neurosci.* 14 1228–1258. 10.3758/s13415-014-0270-2 24683062 PMC4218981

[B10] Carretié L. Kessel D. Carboni A. López-Martín S. Albert J. Tapia M. (2012). Exogenous attention to facial vs non-facial emotional visual stimuli. *Soc. Cogn. Affect. Neurosci.* 8 764–773. 10.1093/scan/nss068 22689218 PMC3791067

[B11] Chartrand T. L. Huber J. Shiv B. Tanner R. J. (2008). Nonconscious goals and consumer choice. *J. Consum. Res.* 35 189–201.

[B12] Crosier B. S. Webster G. D. Dillon H. M. (2012). Wired to connect: evolutionary psychology and social networks. *Rev. Gen. Psychol.* 16 230.

[B13] Cuddy A. J. C. Glick P. Beninger A. (2011). The dynamics of warmth and competence judgments, and their outcomes in organizations. *Res. Organ. Behav.* 31 73–98. 10.1016/j.riob.2011.10.004

[B14] de Jong Gierveld J. van Tilburg T. Dykstra P. (2016). “New ways of theorizing and conducting research in the field of loneliness and social isolation,” in *The Cambridge Handbook of Personal Relationships*, 2nd Edn, eds Vangelisti A. Perlman D. (New York: Cambridge University Press), 1–30.

[B15] DeWall C. N. Maner J. K. (2008). High status men (but not women) capture the eye of the beholder. *Evol. Psychol.* 6 328–341. 10.1177/147470490800600209

[B16] DeWall C. N. Maner J. K. Rouby D. A. (2009). Social exclusion and early-stage interpersonal perception: selective attention to signs of acceptance. *J. Pers. Soc. Psychol.* 96 729–741. 10.1037/a0014634 19309198

[B17] Ellis P. D. (2010). *The Essential Guide to Effect Sizes: Statistical Power, Meta-Analysis, and the Interpretation of Research Results.* Cambridge: Cambridge University Press.

[B18] Engell A. D. Haxby J. V. Todorov A. (2007). Implicit trustworthiness decisions: automatic coding of face properties in the human amygdala. *J. Cogn. Neurosci.* 19 1508–1519. 17714012 10.1162/jocn.2007.19.9.1508

[B19] Ewing L. Rhodes G. Pellicano E. (2010). Have you got the look? Gaze direction affects judgements of facial attractiveness. *Vis. Cogn.* 18 321–330.

[B20] Fiske S. T. Cuddy A. J. Glick P. (2007). Universal dimensions of social cognition: warmth and competence. *Trends Cogn. Sci.* 11 77–83. 10.1016/j.tics.2006.11.005 17188552

[B21] Gardner W. L. Pickett C. L. Jefferis V. Knowles M. (2005). On the outside looking in: loneliness and social monitoring. *Pers. Soc. Psychol. Bull.* 31 1549–1560. 16207773 10.1177/0146167205277208

[B22] Green P. MacLeod C. J. (2016). Simr: an R package for power analysis of generalised linear mixed models by simulation. *Methods Ecol. Evol.* 7 493–498. 10.1111/2041-210X.12504

[B23] Griskevicius V. Kenrick D. T. (2013). Fundamental motives: how evolutionary needs influence consumer behavior. *J. Cons. Psychol.* 23 372–386. 10.1016/j.j.2013.03.003

[B24] Hawkley L. C. Cacioppo J. T. (2010). Loneliness matters: a theoretical and empirical review of consequences and mechanisms. *Ann. Behav. Med.* 40 218–227. 10.1007/s12160-010-9210-8 20652462 PMC3874845

[B25] Hsu S.-M. Pessoa L. (2007). Dissociable effects of bottom-up and top-down factors on the processing of unattended fearful faces. *Neuropsychologia* 45 3075–3086. 17631362 10.1016/j.neuropsychologia.2007.05.019 PMC2045638

[B26] Kenrick D. T. Griskevicius V. Neuberg S. L. Schaller M. (2010). Renovating the pyramid of needs: Contemporary extensions built upon ancient foundations. *Perspect. Psychol. Sci.* 5 292–314. 10.1177/1745691610369469 21874133 PMC3161123

[B27] Khalid S. Deska J. C. Hugenberg K. (2016). The eyes are the windows to the mind: direct eye gaze triggers the ascription of others’ minds. *Pers. Soc. Psychol. Bull.* 42 1666–1677. 27738192 10.1177/0146167216669124

[B28] Kruglanski A. W. Shah J. Y. Fishbach A. Friedman R. Chun W. Y. Sleeth-Keppler D. (2002). “A theory of goal systems,” in *Advances in Experimental Social Psychology*, Vol. 34 ed. Zanna M. P. (San Diego, CA: Academic Press), 331–378.

[B29] Kuznetsova A. Brockhoff P. Christensen R. (2017). lmerTest package: tests in linear mixed effects models. *J. Stat. Softw.* 82 1–26. 10.18637/jss.v082.i13

[B30] Lim M. H. Rodebaugh T. L. Zyphur M. J. Gleeson J. F. M. (2016). Loneliness over time: the crucial role of social anxiety. *J. Abnorm. Psychol.* 125 620–630. 10.1037/abn0000162 27124713

[B31] Lodder G. M. Scholte R. H. Goossens L. Engels R. C. Verhagen M. (2016). Loneliness and the social monitoring system: emotion recognition and eye gaze in a real-life conversation. *Br. J. Psychol.* 107 135–153. 10.1111/bjop.12131 25854912

[B32] MacNamara A. Hajcak G. (2010). Distinct electrocortical and behavioral evidence for increased attention to threat in generalized anxiety disorder. *Depress. Anxiety* 27 234–243. 10.1002/da.20679 20196100

[B33] Maner J. K. DeWall C. N. Baumeister R. F. Schaller M. (2007). Does social exclusion motivate interpersonal reconnection? Resolving the “porcupine problem.” *J. Pers. Soc. Psychol.* 92 42–55. 17201541 10.1037/0022-3514.92.1.42

[B34] Masuda Y. Tadaka E. Dai Y. (2012). Reliability and validity of the Japanese version of the UCLA loneliness scale version 3 among the older population. *J. Jpn. Acad. Community Health Nurs.* 15 25–32. 10.20746/jachn.15.1_25

[B35] Motoki K. Saito T. Nouchi R. Kawashima R. Sugiura M. (2018). Tastiness but not healthfulness captures automatic visual attention: preliminary evidence from an eye-tracking study. *Food Qual. Prefer.* 64 148–153.

[B36] Motoki K. Saito T. Nouchi R. Kawashima R. Sugiura M. (2019). Round faces are associated with sweet foods: the role of crossmodal correspondence in social perception. *Foods* 8:103. 10.3390/foods8030103 30893905 PMC6463122

[B37] Motoki K. Sugiura M. (2017). Consumer behavior, hormones, and neuroscience: integrated understanding of fundamental motives why we buy. *Psychologia* 60 28–43.

[B38] O’Connor J. J. Fraccaro P. J. Pisanski K. Tigue C. C. O’Donnell T. J. Feinberg D. R. (2014). Social dialect and men’s voice pitch influence women’s mate preferences. *Evol. Hum. Behav.* 35 368–375.

[B39] Oosterhof N. N. Todorov A. (2008). The functional basis of face evaluation. 105 11087–11092. 10.1073/pnas.0805664105 PMC2516255 18685089

[B40] Osberg T. M. (1987). The convergent and discriminant validity of the need for cognition scale. *J. Pers. Assess.* 51 441–450. 10.1207/s15327752jpa5103_11 16372844

[B41] Peirce J. W. (2007). PsychoPy—Psychophysics software in Python. *J. Neurosci. Methods* 162 8–13. 17254636 10.1016/j.jneumeth.2006.11.017 PMC2018741

[B42] Pickett C. L. Gardner W. L. (2005). “The social monitoring system: enhanced sensitivity to social cues as an adaptive response to social exclusion,” in *The Social Outcast: Ostracism, Social Exclusion, Rejection, and Bullying*, Vol. 213–226 eds Williams K. D. Forgas J. P. von Hippel W. (New York, NY: Psychology Press).

[B43] R Core Team (2017). *R: A Language and Environment for Statistical Computing.* Venna: R Foundation for Statistical Computing.

[B44] Russell D. W. (1996). UCLA Loneliness Scale (Version 3): reliability, validity, and factor structure. *J. Pers. Assess.* 66 20–40. 8576833 10.1207/s15327752jpa6601_2

[B45] Saito T. Motoki K. Nouchi R. Kawashima R. Sugiura M. (2019). Does incidental pride increase competency evaluation of others who appear careless? Discrete positive emotions and impression formation. *PLoS One* 14:e0220883. 10.1371/journal.pone.0220883 31393917 PMC6687287

[B46] Saito T. Nouchi R. Kinjo H. Kawashima R. (2017). Gaze bias in preference judgments by younger and older adults. *Front. Aging Neurosci.* 9:285. 10.3389/fnagi.2017.00285 28890696 PMC5574931

[B47] Shimojo S. Simion C. Shimojo E. Scheier C. (2003). Gaze bias both reflects and influences preference. *Nat. Neurosci.* 6 1317–1322. 10.1038/nn1150 14608360

[B48] Shin J. E. Kim K. (2019). Loneliness increases attention to negative vocal tone in an auditory Stroop task. *Pers. Individ. Dif.* 137 144–146.

[B49] Sui J. Liu C. H. (2009). Can beauty be ignored? Effects of facial attractiveness on covert attention. *Psychon. Bull. Rev.* 16 276–281. 10.3758/PBR.16.2.276 19293094 PMC3343302

[B50] Todorov A. Baron S. G. Oosterhof N. N. (2008). Evaluating face trustworthiness: a model based approach. *Soc. Cogn. Affect. Neurosci.* 3 119–127. 10.1093/scan/nsn009 19015102 PMC2555464

[B51] Turatto M. Galfano G. (2000). Color, form and luminance capture attention in visual search. *Vis. Res.* 40 1639–1643. 10814751 10.1016/s0042-6989(00)00061-4

[B52] Van Hooff J. C. van Buuringen M. El M’rabet I. de Gier M. van Zalingen L. (2014). Disgust-specific modulation of early attention processes. *Acta Psychol.* 152 149–157. 10.1016/j.actpsy.2014.08.009 25226546

[B53] Williams K. D. Cheung C. K. Choi W. (2000). Cyberostracism: effects of being ignored over the internet. *J. Pers. Soc. Psychol.* 79 748–762. 11079239 10.1037//0022-3514.79.5.748

[B54] Willis J. Todorov A. (2006). First impressions: making up your mind after a 100-ms exposure to a face. *Psychol. Sci.* 17 592–598. 16866745 10.1111/j.1467-9280.2006.01750.x

[B55] Winston J. S. Strange B. A. O’Doherty J. Dolan R. J. (2002). Automatic and intentional brain responses during evaluation of trustworthiness of faces. *Nat. Neurosci.* 5 277–283. 11850635 10.1038/nn816

[B56] Wojciszke B. (2005). Morality and competence in person-and self-perception. *Eur. Rev. Soc. Psychol.* 16 155–188.

[B57] Wojciszke B. Abele A. E. (2008). The primacy of communion over agency and its reversals in evaluations. *Eur. J. Soc. Psychol.* 38 1139–1147.

[B58] Xu M. Li Z. Zhang J. Sun L. Fan L. Zeng Q. (2015). Social exclusion influences attentional bias to social information. *Asian J. Soc. Psychol.* 18 199–208. 10.1016/j.jbtep.2017.11.004 29136514

[B59] Zhang Y. Risen J. L. (2014). Embodied motivation: using a goal systems framework to understand the preference for social and physical warmth. *J. Pers. Soc. Psychol.* 107 965–977. 10.1037/a0038153 25437131

[B60] Zumbo B. D. Hubley A. M. (1998). A note on misconceptions concerning prospective and retrospective power. *Statistician* 47 385–388. 10.1111/1467-9884.00139

